# Exploring the Association between Alzheimer’s Disease, Oral Health, Microbial Endocrinology and Nutrition

**DOI:** 10.3389/fnagi.2017.00398

**Published:** 2017-12-01

**Authors:** Alice Harding, Ulrike Gonder, Sarita J. Robinson, StJohn Crean, Sim K. Singhrao

**Affiliations:** ^1^Dementia & Neurodegenerative Diseases Research Group, Faculty of Clinical and Biomedical Sciences, School of Dentistry, University of Central Lancashire, Preston, United Kingdom; ^2^Nutritionist, Freelance Science Writer, Hünstetten, Germany; ^3^Faculty of Science and Technology, School of Psychology, University of Central Lancashire, Preston, United Kingdom

**Keywords:** Alzheimer’s disease, co-morbidities, diet, endocrine microbiomes, periodontitis

## Abstract

Longitudinal monitoring of patients suggests a causal link between chronic periodontitis and the development of Alzheimer’s disease (AD). However, the explanation of how periodontitis can lead to dementia remains unclear. A working hypothesis links extrinsic inflammation as a secondary cause of AD. This hypothesis suggests a compromised oral hygiene leads to a dysbiotic oral microbiome whereby *Porphyromonas gingivalis*, a keystone periodontal pathogen, with its companion species, orchestrates immune subversion in the host. Brushing and chewing on teeth supported by already injured soft tissues leads to bacteremias. As a result, a persistent systemic inflammatory response develops to periodontal pathogens. The pathogens, and the host’s inflammatory response, subsequently lead to the initiation and progression of multiple metabolic and inflammatory co-morbidities, including AD. Insufficient levels of essential micronutrients can lead to microbial dysbiosis through the growth of periodontal pathogens such as demonstrated for *P. gingivalis* under low hemin bioavailability. An individual’s diet also defines the consortium of microbial communities that take up residency in the oral and gastrointestinal (GI) tract microbiomes. Their imbalance can lead to behavioral changes. For example, probiotics enriched in *Lactobacillus* genus of bacteria, when ingested, exert some anti-inflammatory influence through common host/bacterial neurochemicals, both locally, and through sensory signaling back to the brain. Early life dietary behaviors may cause an imbalance in the host/microbial endocrinology through a dietary intake incompatible with a healthy GI tract microbiome later in life. This imbalance in host/microbial endocrinology may have a lasting impact on mental health. This observation opens up an opportunity to explore the mechanisms, which may underlie the previously detected relationship between diet, oral/GI microbial communities, to anxiety, cognition and sleep patterns. This review suggests healthy diet based interventions that together with improved life style/behavioral changes may reduce and/or delay the incidence of AD.

## Introduction

Alzheimer’s disease (AD) is the most common form of dementia, constituting 60%–80% of all cases. Due to the rising number of dementia cases and the paucity of adequate treatment for AD, emphasis for future management of this disease is on identification and modification of potential risk factors for AD onset and progression.

## Alzheimer’s Disease

There are two forms of AD: the inherited form, with known genetic factors, and the sporadic form, with an unknown cause. The sporadic form is the focus of this manuscript, as the vast majority of AD cases fall into this category. Whatever the form of AD, the overall condition presents clinically with depression and deteriorating cognition and at post mortem with two neuropathological hallmark lesions. These lesions are proteins represented as senile plaques composed of amyloid-beta (Aβ), and hyperphosphorylated tau protein coated neurofibrillary tangles. For the sufferer, the course of disease is relentless, and families shoulder the emotional impact.

While clinically AD is described as “irreversible memory loss” a recent clinical study of the early intervention MEND™ protocol demonstrated reversal of the functional loss in the prodromal AD cases (Bredesen et al., 2016). In this study a small cohort of patients re-gained memory by avoiding risk factors as suggested in the MEND™ protocol (Bredesen et al., 2016). The MEND™ protocol represents proof of concept for modifiable risks in AD, and supports the concept of risk factor identification and modification to delay/slow down AD.

**Figure 1:**
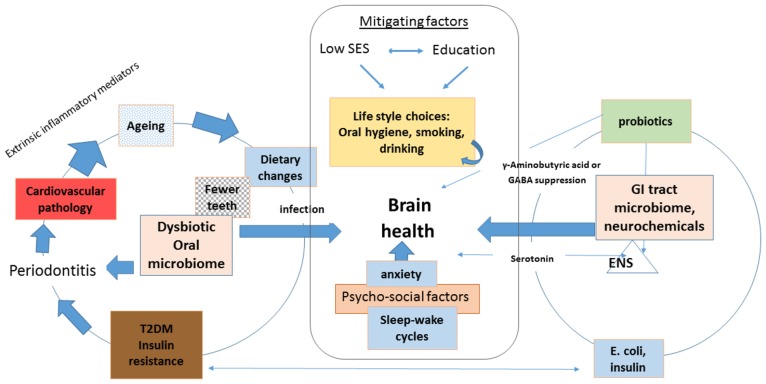
Schematic to show the knock on effect from an oral condition such as periodontitis to the development of co-morbid states in the order of periodontal disease cardiovascular and Alzheimer’s disease (AD) with input from the metabolic disorder like type 2 diabetes (T2DM). These conditions disturb mental health. Mitigation through educational programmes and improved lifestyles involving better oral hygiene, cessation of smoking, nutritional intervention especially prebiotics providing a balance of neurochemicals from the GI-tract-brain-axis. Together with dental intervention, diet and change in life styles potentially will provide improved cognition. GI tract microbiome having input on the brains health through microbial changes whereby γ-aminobutyric acid (GABA) deficit affects behavior. Abbreviations: ENS, enteric nervous system; SES, socio-economic status; GI, gastrointestinal.

Previous research suggests that people of a lower socio-economic status (SES) and those with a poor education level and women (Brayne and Calloway, 1990; Chen and Miller, 2013; Russ et al., 2013) have poorer cognitive functioning in later life (Marden et al., 2017). This group of individuals would appear to be most at risk of AD development. Recently, the FINGER trial study (Rosenberg et al., 2017) demonstrated better cognition following multi-domain lifestyle intervention in the elderly who were at risk for developing dementia due to low SES and limited education. In the Rosenberg et al. (2017) study, intervention supported changes to ensure a healthy diet, exercise, cognitive training and management of vascular risk factors. The overall outcome was encouraging (Rosenberg et al., 2017); however, oral health status was omitted from their multi-domain risk factor analysis. One pathway between low SES and premature mortality is the adoption of unhealthy behaviors, such as smoking and drinking (Krueger and Chang, 2008; Singh et al., 2013). These life style behaviors increase periodontal disease manifestation (Borrell et al., 2006; Persson, 2008; Bonfim et al., 2013) and affect diseases that are co-morbid with AD (Figure 1).

Dietary choice, in healthy individuals, primarily depends on the state of a person’s dentition (chewing ability) as well as on their appetite and craving for a specific type of food. It is proposed that the microbial endocrine signaling between the host and gastrointestinal (GI) tract microbiome neurochemicals also influence appetite related decision-making (Lyte, 2013, 2014). Therefore, another pathway to support the importance of behavioral change via the dysbiotic GI tract microbiome is from a lack of γ-aminobutyric acid (GABA) expressing bacteria (Foster et al., 2016; see Figure 1). The healthy brain is abundant in GABAergic neurons, and the host’s neurotransmitters are known to play diverse roles in regulating an individual’s behavior (Brown et al., 2008), and to some extent a holistic wellbeing. Another shared behavioral trait between microbial endocrinology and human GABA is sleep (Brown et al., 2008; Galland, 2014, see sleep section). The overall aim of the current article is to explore the underlying mechanisms behind the risk factors identified from both periodontal disease pathogens and GI tract microbiome neuroendocrine mediated behavioral aspects. Together they may show potential for further improvements in cognition if a healthy diet, better oral health and a symbiotic GI microbiome resumes.

## Periodontal Disease

Periodontal disease refers to a group of oral (aggressive and chronic) diseases with polymicrobial etiology affecting the tooth supporting tissues that result from a host’s immune dysbiosis (Olsen et al., 2017). It is clear that some genetic influence exist in the likelihood of periodontal disease development (Genco and Borgnakke, 2013), however, psychosocial and environmental factors appear more important in its progression. In addition, psychosocial factors can influence disease onset and progression, with those reporting higher levels of anxiety being more susceptible to periodontal disease (Cekici et al., 2014). Anxiety also contributes to endogenous inflammation (Kim and Jeon, 2017), and by reducing inflammation and oxidative stress levels of periodontal disease (Waddington et al., 2000), could guard against AD (Figure 1).

### Mechanisms Linking Alzheimer’s Disease with Periodontitis

The oral risk factors link to periodontal pathogens accessing the brain (Riviere et al., 2002; Poole et al., 2013). These environmental factors may have contributed to dementia development in the elderly. Several studies have supported the links between life-style choices, specifically engaging with oral health practices and AD development (Kondo et al., 1994; Stein et al., 2007; Paganini-Hill et al., 2012; Luo et al., 2015; Ide et al., 2016). More recently, a Taiwanese study found a strong link between chronic periodontal disease (exposures of around 10-year period) and AD (Chen et al., 2017). This retrospective study (Chen et al., 2017) correlates with a previous prospective study in which circulating antibodies to two oral bacteria were linked to cognitive deficit 10 years later (Sparks Stein et al., 2012). SES (poor oral health, smoking, unhealthy diet) factors are very important when considering infections of the brain. According to literature, periodontal disease does contribute to systemic markers of inflammation (Loos, 2005). Research has shown that systemic inflammatory markers mediate the development of AD (Schmidt et al., 2002; Holmes et al., 2003; Engelhart et al., 2004; Kamer et al., 2009; Sparks Stein et al., 2012). This finding is the crucial link that explains the association of periodontal disease with both the etiology of cardiovascular pathologies (Libby et al., 2002), and insulin resistance (Craft, 2005). Rather surprisingly, *Escherichia coli* K12 possesses insulin that is similar to the mammalian functional protein (LeRoith et al., 1981). The fact that *E. coli* lipopolysaccharide (LPS) in AD brains co-localizes with Aβ hallmark protein (Zhan et al., 2016; Zhao et al., 2017) suggests extrinsic sources of insulin have the potential to contribute to excess insulin and/or its resistance development. It may be plausible to suggest that microbial endocrinology plays a role in the development of diabetes in the AD brain.

### Shared Risk Factors for Alzheimer’s Disease and Periodontitis

#### Aging

Periodontal disease can start early in life and can progress to chronic periodontitis in the 40–50 year age range (Schätzel et al., 2009). Studies show a greater severity of disease in older populations (Tawse-Smith, 2007; Chen et al., 2017) and an explanation could be related to the periodontal pathogens becoming easily “radicalized” or dysbiotic (Harding et al., 2017) due to dietary changes. For example, *P. gingivalis* is able to change its lipid A phosphate composition within its LPS macromolecule, in response to differing environmental conditions (paucity of micronutrient bioavailability). This can produce lipid A structures that can differentially activate Toll-like Receptor 4 (TLR 4; Al-Qutub et al., 2006) creating an inflammophilic environment for selection of co-species by dysbiosis and controlling microbial competition. Incidence of AD exponentially rises with age from 3% among the 65–74-year age group to almost 50% among those around 85 years and older.

#### Diet

Tooth loss may be associated with nutritional deficits as it does influence food selection (Ship et al., 1996). It is conceivable that omission of fresh vegetables and fruit from the diet is likely due to difficulties in biting and chewing, whatever the age that tooth loss occurs. Tooth loss could therefore lead to a reliance on comfort foods, rich in carbohydrates and fats that are associated with an increased risk of cardiovascular disease and stroke. Vascular risk factors are important in the etiology of dementia, and loss of teeth has been associated with an increased risk of stroke (Joshipura et al., 2003), coronary heart disease (Loesche et al., 1998) and hypertension (Taguchi et al., 2004) and memory loss in later life (Kondo et al., 1994).

#### Genetic Predisposition

AD has an inherited early-onset form (O’Brien and Wong, 2011): the late onset form also has high heritability but is also associated with numerous pathogens, many of which are able to promote the key features of AD in laboratory models. Interestingly, AD related genes from Genome Wide Association Studies overlap with the host genes employed by several pathogens, including *P. gingivalis*, suggesting that susceptibility genes and pathogens may condition each other’s disease promoting effects (Carter, 2017).

#### Infection

Periodontal disease is of known polymicrobial etiology, and patients who eventually suffer from sporadic AD tend to have repeat infections before their clinical diagnosis of dementia (Dunn et al., 2005). This is supported by the finding of microbes in post mortem AD brains (Riviere et al., 2002; Miklossy, 2011, 2015; Emery et al., 2017).

#### Inflammatory Signaling

An inflammophilic environment is highly desirable for oral commensals to become pathogenic and to support periodontal disease initiation and progression under the influence of *P. gingivalis*. The LPS of *P. gingivalis* shows heterogeneity in the lipid A region, giving rise to two isoforms designated LPS_1435/1449_ and LPS_1690_ (Herath et al., 2013). These isoforms are responsible for specific immune signal transduction pathways (NF-κB; p38 MAPK and/or ERK1/2) that differentially activate cells from different linages (Darveau et al., 1998; Reife et al., 2006; Ding et al., 2013; Herath et al., 2013). However, the plausibility of the theory that *P. gingivalis* LPS orchestrates differential immune signaling for initiation of distant organ specific inflammatory pathology also lies in this endotoxin in relation to cytokine secretion (Darveau et al., 1998). Consuming omega-3 fatty acids of marine origin (DHA, EPA), polyphenols and other non-nutritive compounds of fish, vegetables, nuts and fruit act as anti-inflammatory agents (Calder et al., 2009).

#### Socio-Economic Status and Low Education

An association between periodontal disease, low SES and AD has been established (Brayne and Calloway, 1990; Borrell et al., 2006; Persson, 2008; Bonfim et al., 2013). Exactly how and why SES is a risk factor for AD is unclear but the assumption is that SES influences environmental factors, such as smoking and alcohol habits (Brayne and Calloway, 1990; Russ et al., 2013). However, Rosenberg et al. (2017) were unable to link sex, age and education (low SES) to poor cognition in their multi-domain lifestyle interventional study. One potential reason Rosenberg et al. (2017) did not find a link between SES risk factors and AD could be that they did not include oral health in their intervention. Oral health would have been useful to include, as low SES shows associations with periodontal disease manifestation through harboring higher proportions of oral bacteria with greater virulence. In addition, excessive smoking and consumption of alcohol is associated with lower SES and both of these adverse health behaviors can have a negative effect on oral hygiene (Singh et al., 2013). People who have a lower SES may also lack the income to seek dental treatment and purchase oral hygiene measures, rather than it being through a lack of education.

Research has discovered that low education achievement can lead to higher levels of periodontal disease, even when adjusting for age, gender and SES (Borrell et al., 2006). This suggests that being less educated may be a risk factor, independent of SES background of the individual. One potential reason for the link between education level and severe periodontitis may be that those with a lower level of educational achievement may receive less information and education regarding how to maintain their oral health (Park et al., 2016).

#### Exercise

Chronic inflammation and oxidative stress are major biological changes seen inherently in the aging process (Cannizzo et al., 2011). It is suggested that regular exercise has anti-inflammatory and anti-oxidant effects (Taafe et al., 2000). Regular exercise and nutrition appear to link with better periodontal health (Bawadi et al., 2011) and generally through lowering the levels of peripheral inflammatory markers (Taafe et al., 2000).

#### Psychomicrobiomics

The GI tract microbiome-brain axis conceptualizes microbial endocrine contribution as having an influence on emotional behavior in humans and laboratory animals (Bravo et al., 2011). Microbes appear to have neurohormones/neurotransmitters that function in a similar way to those of human hosts. Germ-free mice are a good model for understanding the GI microbial endocrinology in relation to stress and stress related hormones (Sudo et al., 2004). After subjecting germ-free mice to a battery of psychological testing procedures, they displayed reduced anxiety-like behavior. Transfer of strain-matched microbes reversed this phenotype in germ-free mice at an earlier age (Diaz Heijtz et al., 2011; Clarke et al., 2013). This shows the importance of symbiotic relationships of the host with its microbiomes. Maintaining a balance of these microbiomes is important and achievable through a dietary intake that supports neurotransmitters/neurohormones and so balances local activities and maintains health of the host.

### Mediating Factor: Gastrointestinal Tract Microbiome

The oral cavity is important for a number of nutritional functions that are required for a healthy existence and, as such, links it to the GI tract. As noted previously, the GI tract microbiome can affect AD independently of periodontal disease. However, the GI tract microbial challenges also pose an interesting analogy through the dysbiotic microbiome concept to the intestinal mucosal barriers becoming permeable (Daulatzai, 2014).

The hypothalamic-pituitary-adrenal (HPA) axis responds to periodontal disease polymicrobial infections. Related inputs from the CNS may modify GI tract function, while inputs from the alimentary canal can modulate symptom processing via sensory pathways to the brain. Since AD onset progresses slowly to the clinical expression, Brandscheid et al. (2017) examined whether the asymptomatic neuropathology correlated with changing bacteria in the GI tract of a familial (5xFAD) mouse model. Interestingly the study found reduced protein breakdown and this was related to poor trypsin secretions. Lower levels of trypsin affected this niche as demonstrated by a changing fecal microbial composition in an age-dependent manner (Brandscheid et al., 2017). The consequences of a changing gut microbiome are that specific bacterial species taking up residency may acquire the potential to break down mucosal barriers.

### Mediating Factor: Insulin Resistance and Inflammation

The most important underlying metabolic disturbances that raise the risk for both AD and periodontal disease are insulin resistance and a state of chronic inflammation. Based on the variability in the inflammatory component of AD cases, three main subtypes of this disease are described, which are distinguishable by metabolic profiling. Two of these are described as the “inflammatory” type and “non-inflammatory” type (Bredesen, 2015). The “inflammatory” type often encompasses an ApoE4 allele carrier, which presents with markers of systemic and cerebral inflammation such as C-reactive protein (CRP) and IL-6 levels, insulin resistance and hyperhomocysteinemia. In the “non-inflammatory” type AD insulin resistance, hypovitaminosis D, hyperhomocysteinemia and reduced hormones dominate while indicators of systemic inflammation are absent. This does not however exclude the existence of intra-cerebral inflammation. Since the inflammatory and non-inflammatory subtypes are insulin resistant, and often FDG-PET scans show temporal parietal reductions in glucose utilization (Cunnane et al., 2011; Bredesen, 2015), their management by nutritional support is plausible.

### Diet Based Intervention to Support Mixed-Pathologies

Good nutrition plays an important role in health. The five leading global risks for mortality appear to be high blood pressure, tobacco usage, hyperglycemia, physical inactivity and obesity. Nutrition can address three of these five global risks successfully. For example, if individuals consume low amounts of plant-derived foods, they are at increased risk of developing T2DM, metabolic syndrome, some cancers, and AD (Salas-Salvadó et al., 2016). Although a relationship between nutrition and AD onset is proposed, the mechanisms, which may explain how poor nutrition leads to AD, are incomplete. Here we explore how poor nutrition may not only act as a risk factor for poor oral health, but may also influence the mechanisms that mediate the relationship between periodontal disease and AD development. Furthermore, we explore interventions that can support good nutrition.

### Risk Factor: Western Diet

The MEND™ protocol is diet based, and shows reversal of the functional loss in the prodromal AD cases (Bredesen et al., 2016). This study refers to a cohort (*N* = 10) of patients who were able to re-gain memory by avoiding risk factors as suggested in the MEND™ protocol (Bredesen et al., 2016). However, it is unclear if all the foods found within a Western diet directly disturb brain function, or if it is single nutrients or a combination of different, dietary elements that have an adverse impact on brain health. For example, there is no strong argument (Ravnskov et al., 2014; Harcombe et al., 2016), for avoiding saturated fats, however, when combined with high glycaemic carbohydrates, saturated fats do have negative health consequences (Varela-López et al., 2016). However, the prospective PURE cohort study in 18 countries from five continents found that total fat intake and individual types of fatty acids are associated with a decreased risk of total mortality, and saturated fat intake correlates inversely with risk for strokes, an established risk factor for AD (Dehghan et al., 2017).

Diet also has an impact on periodontal disease, an established risk factor for AD (Kamer et al., 2015; Chen et al., 2017; Harding et al., 2017). Therefore, promoting a healthy diet that supports good oral health is an important preventative measure for AD. A recent epidemiological study on a Japanese population showed a more advanced periodontal disease in people who had a lower fat intake (Hamasaki et al., 2017). The authors concluded that a diet low in fat and high in carbohydrates might be associated with a higher prevalence of periodontal disease. On the other hand, the consumption of vegetables, fruit, β-carotene, vitamin C, β-tocopherol, EPA and DHA helped patients to reduce their levels of periodontal disease (Dodington et al., 2015).

For the prevention of insulin resistance and inflammation, diets high in monounsaturated (e.g., olive oil, avocados, macadamias, chicken fat) and omega-3 polyunsaturated fatty acids of marine origin are recommended, e.g., a Mediterranean style diet (Schwingshackl and Hoffmann, 2014). Epidemiological and interventional studies have repeatedly shown superior outcomes in weight loss (Hession et al., 2009), weight maintenance (Larsen et al., 2010), insulin resistance, triglyceride lowering and HDL cholesterol raising (Shai et al., 2008; Bazzano et al., 2014), and lowering of CRP (Bazzano et al., 2014) with lower carbohydrate diets containing more protein and fat. The same applies for the adoption of a Mediterranean type diet, which by definition is not low in fat (Schwingshackl and Hoffmann, 2014). The PREDIMED intervention trial in individuals with elevated cardiovascular risk compared a Mediterranean eating pattern supplemented with olive oil or nuts with a lower fat diet. The results showed significantly lower numbers of cardiovascular events in those who ate the diets supplemented with nuts or olive oil (Estruch et al., 2013) and slower cognitive decline (Estruch et al., 2013; Martínez-Lapiscina et al., 2013). In addition, foods high in important micronutrients, such as omega-3 fatty acids, vitamins C, D, E, B12 and B6, zinc, iron, iodine and magnesium, potassium, magnesium and dietary fiber can support brain health (Cunnane, 2006).

Beside their negative impact on insulin resistance and inflammation, diets high in simple carbohydrates may impair the integrity of the BBB (Hsu and Kanoski, 2014). Compromised BBB function in individuals susceptible to periodontitis will allow microbes to enter the brain and this could increase the chances of developing AD. Adopting a healthier Mediterranean style diet (Schwingshackl and Hoffmann, 2014), or eating fish, vegetables, nuts, cocoa and a variety of berries (Poulose et al., 2014), which are rich in antioxidants and have anti-inflammatory properties, may help to reduce gingival inflammation. Therefore, a diet, which reduces inflammation and insulin resistance, may help to maintain oral and brain health and thus may reduce the risk of AD (Singh et al., 2014).

### Risk Factor: Vitamins

Vitamins are an important element of a well-balanced diet and vitamin deficits can have important consequences for both periodontal disease and cognitive performance. Epidemiological studies show that low serum levels of 25-hydroxyvitamin D (25OHD) are associated with increased risk of dental caries and periodontal disease (as well as other risk factors such as cardiovascular diseases, T2DM and depression), which, in turn, are risk factors for developing AD (Grant, 2009; Toffanello et al., 2014a,b). Therefore, monitoring vitamin D levels in populations such as the elderly, who are at risk of AD, is important. Some individuals may be at a greater risk to a vitamin D deficiency because of their dietary choices. For example, vegans could have lower intakes because they avoid vitamin D rich food such as fatty fish and free-range eggs, or individuals who live in climates where exposure to the sun (UV-B radiation) is scarce throughout the year. More importantly, mature vitamin D results from the pro-hormone synthesis initiated by UV-B radiation from the sun and the ability to do so declines during aging. Properly targeted nutrition, careful sun exposure and supplements can help to achieve health-supporting values of 25OHD ≥75 nmol/l.

Vitamin B also appears to be important for protecting against the onset and progression of AD. Inadequate levels of B vitamins (folate, B12, B6) relate to cognitive impairment and incidence of dementia in the elderly (Morris, 2012), possibly because lower levels of these micronutrients elevate plasma homocysteine (HCys). HCys is a neuro and vasotoxic metabolite that may lead to impaired S-adenosylmethionine-dependent methylation reactions, which is vital to CNS functioning (Selhub et al., 2010; Köbe et al., 2016). Even without clinically manifested deficiency of vitamin B12, levels in the lower normal range were associated with poorer memory performance and a reduced microstructural integrity of the hippocampus, a brain area, typically affected first in AD (Köbe et al., 2016).

Foods like meat, eggs and fish provide sufficient amounts of vitamin B12 to the body (Cunnane, 2006). Strict vegetarians on plant foods lacking in B12 must consider supplementing their diet with B12. However, interventional studies with single or few nutritional supplements have yielded mixed results (Eussen et al., 2006; Cunnane et al., 2009; Quinn et al., 2010; Yurko-Mauro et al., 2010) and one explanation for this discrepancy maybe that in the elderly, methylation reactions are less effective. Therefore, some researchers recommend offering methylated folate and cobalamin to seniors to raise vitamin B12 or lower homocysteine blood levels (Bredesen, 2014; Hara et al., 2016). In a recent prospective case-control study, Hara et al. (2016) found patients with AD or related diseases who took a supplement containing L-methylfolate, methylcobalamine and N-acetyl-cysteine, showed a noticeable benefit. The authors suggest that the methylated vitamins are fully reduced and in bioactive functional form to combat hyperhomocysteinaemia (Hara et al., 2016). Another nutritional formulation containing sulfur containing N-acetyl-cysteine and S-adenosyl-methionine, in addition to vitamins, also showed some promise in slowing the cognitive decline seen in prodromal AD cases (Remington et al., 2015). In summary, careful monitoring of vitamin D and B levels, and dietary interventions as required, may help to prevent the onset and progression of AD. Additional research is needed to identify other vitamins and nutrients, which can support oral health and preserve cognitive functioning.

### Risk Factor Poor Gastrointestinal Tract Health: Pre/Probiotics

Recently attention has focused on the gut-brain axis and the role of probiotics in maintaining both physical and mental health. Diets rich in fruit and vegetables and low in meat are associated with a greater prevalence of *Prevotella* species rather than *Bacteroides* species, both of which express genes for different digestive enzymes (Greiner et al., 2014). A vegetarian diet has been shown to reduce gut inflammation, increasing commensal microbes and decreasing pathobionts (Kim et al., 2013). This in turn reduces intestinal lipocalin-2, an adipokine, associated with inflammation and insulin resistance development (Zhang et al., 2008; Kim et al., 2013).

Supplementation with probiotics appeared to be useful in the maintenance of oral health (Krasse et al., 2006); with the daily consumption of a probiotic milk drink reducing plaque induced gingival inflammation (Slawik et al., 2011). Furthermore, Riccia et al. (2007) have shown the probiotic *Lactobacillus brevis* to have an anti-inflammatory effect on patients with chronic periodontitis. However, a recent systematic review concluded that current probiotics based treatments for periodontal disease only produce short-term benefits (Jayaram et al., 2016; Bakarcic et al., 2017). Therefore, further research regarding whether the long-term prophylactic use of probiotics is useful to support oral health or whether probiotics to treat active periodontal disease is desirable.

Probiotics may also reduce the onset of AD by stimulating the production of GABA, the inhibitory neurotransmitter in the CNS, via glutamate signaling (Bhattacharjee and Lukiw, 2013). This is significant as studies performed on post mortem brain tissues show reduced GABA concentrations in AD patients. In addition, a recent randomized, double blind, controlled, clinical trial showed that consumption of a mixture of probiotics over a 12-week period had a positive effect on cognitive function in AD patients (Pistollato et al., 2016). Probiotics decrease the levels of proinflammatory cytokines IL-1β, IL-5, IL-6, IL-8 and TNF-α, which are upregulated in the elderly, and increase numbers of activated lymphocytes, natural killer cells and phagocytosis (Rincon et al., 2014; Wang et al., 2015). All these factors indicate an improvement in the adaptive immune response, along with a reduction in inflammation. Overall, it would appear that probiotics have the potential to provide a beneficial effect on cognitive decline and oral health. The fact that probiotics show an anti-inflammatory effect, along with the established GI tract-brain axis, would provide a possible explanation to this positive effect and suggest a potential mechanism for a reduction in onset of AD (Pistollato et al., 2016).

### Risk Factor: High Blood Glucose

Not only do patients with T2DM and other states of insulin resistance show a higher risk of periodontitis, but they also show a higher risk for AD (Ahmed et al., 2017; Górska et al., 2017). In addition, high blood glucose emerges as a risk factor for both diseases (Crane et al., 2013; Miranda et al., 2017). Crane et al. (2013) reported a study of a large cohort of seniors without dementia over a period of 6.8 years. After adjusting for potential confounding variables, participants with higher blood glucose readings in the preceding years had a significantly increased risk of dementia (Crane et al., 2013). The negative impact of high glucose levels has implications for non-diabetic elderly individuals who have higher glucose and HbA1c levels on memory performance, hippocampal volume and microstructure (Kerti et al., 2013). Therefore, strategies aimed at lowering glucose levels, not only in diabetics but also individuals with blood glucose within in the normal range, may beneficially influence cognition and oral health in the older population. The best way to achieve optimal blood glucose levels is to reduce the intake of highly processed carbohydrate rich foods, high glycaemic carbohydrates and to increase fiber, fat and protein (Volek et al., 2009).

### Risk Factor—Poor Glucose Utilization

Lack of brain insulin or the intact functioning of the insulin signaling cascade leads to problems with Aβ clearing, learning and memory, satiety and regulation of feeding behaviors. Additionally, disturbed glucose utilization is seen as part of the normal aging process (Yao et al., 2011). Fortunately, the elderly, including those with mild cognitive impairment and mild forms of AD, are able to take up ketone bodies, like β-hydroxybutyrate (BHB) and acetoacetate, through insulin independent monocarboxylate transporters (Cunnane et al., 2016). Ketone bodies serve as an alternative and efficient fuel for neurons and other brain cells, as well as protecting neurons from oxidative damage. Moreover, the ketone body BHB is able to raise the levels of brain derived neurotrophic factor (BDNF), which is important for growth of new neurons in the hippocampus (Cunnane et al., 2016; Kullmann et al., 2016).

Ketone bodies are metabolites of fatty acid oxidation. The liver is the main peripheral organ to produce ketone bodies, but the astrocytes in the brain are also able to build ketones from medium chain fatty acids (Nonaka et al., 2016; Thevenet et al., 2016), which are saturated fatty acids with chain lengths of 8–12 carbon atoms (C8–C12). Research on healthy individuals has shown that small amounts of medium chain triglycerides (MCTs, 20–30 g C10/d) are able to close an energy gap typically found in aging individuals (Courchesne-Loyer et al., 2013) and delay the onset of AD symptoms (Reger et al., 2004). The achieved degree of ketonemia (ca. 0, 5 mmol/l) was estimated to contribute to up to 9% of brain energy metabolism. For higher ketone body levels it would be necessary to eat more MCTs, adopt a strict diet (very low-carbohydrate ketogenic diet) or to fast.

As MCTs, such as those found in virgin coconut oil, are able to raise ketone body levels mildly, even in the presence of an ordinary diet, they offer an easy way to achieve supply of alternative fuel for neurons with compromised glucose utilization. In a case study, Newport et al. (2015) reported improvements in a patient with early onset AD using coconut oil, MCTs and ketone supplements. As even the healthy aging brain shifts from glucose-driven bioenergetics towards a compensatory ketogenic pathway (Yao et al., 2011), it seems sensible to use ketogenic foods (high quality, natural fatty foods and fats) to reduce the risk of cognitive decline as a result of energy deficiencies and insulin resistance. Although coconut oil raises hepatic ketone levels only mildly, it appears beneficial and potentially helpful for astrocytes to synthesize ketone bodies and then deliver them to neighboring neurons. A study with astrocytes in culture demonstrated that lauric acid (C12), the major medium chain saturated fatty acid constituent of coconut oil, is able to cross the BBB and activate ketogenesis in astrocytes (Nonaka et al., 2016). Alternatively, oil pulling (whereby oil is held in the mouth) with coconut oil was able to reduce the *S. mutans* populations in saliva, and this could be a means to aid the maintenance of oral health (Kaushik et al., 2016). A preliminary report by Peedikayil et al. (2015) described the effects of oil pulling with coconut oil, which contains a high proportion of the anti-inflammatory and antimicrobial compound lauric acid. It led to a decrease in plaque formation and improved gingival indices after 30 days of its application in African adolescents.

## Life Style Choices

### Risk Factor: Smoking

Smoking tobacco increases the severity with which periodontal disease progresses. For example, the use of nicotine is shown to select for periodontal pathogens, including *P. gingivalis*, *Treponema denticola* and *Tannerella forsythia*, which will encourage progression of this oral disease (Zambon et al., 1996). The periodontal indices change dramatically, as smoking not only leads to harboring higher proportions of oral bacteria that colonize rapidly, but also encourages colonization of species of the more virulent, red complex pathogens such as *T. forsythia* and *P. gingivalis* strains than not smoking (Kamma et al., 1999). The highly virulent forms of *T. forsythia* and *P. gingivalis* in greater numbers are capable of maintaining severe inflammation, and their invasive property continues to exacerbate pathology of the periodontium (Kamma et al., 1999; Bergström, 2004). The inflammation arising from the microbes which result from poor hygiene along with secondary mediators from bone regulatory factors (Takahashi, 2005; Olsen et al., 2016) and the innate immune response in smokers ultimately succumb to a higher rate of alveolar bone erosion (Grossi et al., 1995). Therefore, inflammatory burden due to smoking (Kamma et al., 1999) can undermine the developing chronicity of periodontitis alongside the impact on health of the distant organs affected by periodontal pathogens.

### Risk Factor: Drinking Alcohol

Drinking alcohol can lead to a moderate increase in the risk of periodontal disease (Tezal et al., 2004; Genco and Borgnakke, 2013). High alcohol consumption affects the innate immune system in a negative manner; with chronic alcohol consumption being predictive of higher levels of inflammatory markers (McDade et al., 2006; Barr et al., 2016). Conversely, drinking alcohol in moderation is thought to reduce periodontal disease, by a positive effect on the innate immune system, and so, reduce the risk of vascular diseases, and AD. In addition, moderate amounts of wine (Evers et al., 2009) can have beneficial anti-inflammatory effects and so may reduce the risk of developing chronic diseases, such as periodontitis and AD.

### Risk Factor: Poor Sleep

Recent research by Sprecher et al. (2017) has highlighted the link between poor sleep and AD development. The study found higher levels of pre-clinical AD biomarkers in cerebrospinal fluid samples in people reporting several measures of poor sleep such as higher levels of daytime somnolence. Therefore, attending to good sleep hygiene is an important preventative measure against AD development (Harding et al., 2017). Specifically, adequate sleep in advancing age is important for retaining memory, and for the effective functioning of the glymphatic system mediated cleansing of the brain (Iliff et al., 2013). The HPA axis has been shown to play a role in the maintenance of sleep/wake patterns. Infections from dysbiotic microbiomes including the GI tract microbiome will modulate the HPA through changes in the neurochemicals and inflammatory mediators (Galland, 2014). For example, proinflammatory cytokines are potent inducers of non-rapid eye movement (nREM) sleep (Galland, 2014). Deterioration of the circadian system that controls sleep/wake cycles in the elderly may contribute to the excess accumulation of abnormal proteins in AD brains and impairments to the glymphatic cleansing system (Yesavage et al., 2003). Active periodontal disease and reduced glymphatic system functioning, due to restricted sleep periods, can also impair the brain’s ability to clear microbes, such as *P. gingivalis* (Harding et al., 2017). Furthermore, there is some evidence to suggest *P. gingivalis*, the keystone pathogen of chronic periodontitis, accesses the brains of experimental mice (Poole et al., 2015; Singhrao et al., 2017), and can disturb the microglial cell day/night activity (Takayama et al., 2016). This implies that *P. gingivalis* infection through chronic periodontitis can further contribute to poor quality sleep.

Humans are diurnal mammals and hence many of their metabolic activities have evolved for activity and storage during the day, and rest, repair and use of stored nutrients during the night. This is supported by hormonal rhythms, like the melatonin rhythm, which is mainly regulated by light and heavily disturbed by blue wavelength emitting devices like smartphones, tablets, TV screens and e-readers (Heo et al., 2017). The daily rhythms make us more insulin resistant in the evening and during the night (Carrasco-Benso et al., 2016; Morris et al., 2016), therefore large dinners, especially those with a heavy carbohydrate load should be avoided in the evening when the body is meant to fast and to use stored fats. In addition, a heavy dinner can disturb sleep and impair sleep quality. Therefore, it is recommended that people fast for at least 3 h before going to bed and to reduce the carbohydrate content, especially of the evening meal (Bredesen, 2014).

Other dietary interventions found to help promote sleep include, avoiding caffeine-containing foods and drinks, if not well tolerated, before bedtime, and alcohol, which can increase sleepiness but disturbs regenerative deep sleep phases (Ebrahim et al., 2013; Clark and Landolt, 2017). Alternatively, eating cherries or kiwifruits can promote sleep via their naturally high levels of melatonin (Pigeon et al., 2010; Lin et al., 2011). Drinking milk before bed may also increase drowsiness due to its high level of tryptophan and B vitamins (Peuhkuri et al., 2012). Finally, exercise taken during the day can have a positive impact on sleep, especially when undertaken outside in natural daylight (Reid et al., 2010).

## Future Perspectives

It is important to recognize the impact of environmental factors on the mental health outcomes of the elderly. Specifically, we argue that nutrition and subsequent impact on oral health, often overlooked should be a priority. Although, oral health may be less of a priority, when compared to the management of other conditions associated with old age, it is clear that poor oral health may cause or aggravate other conditions such as T2DM, which in turn could be the risk factors in AD development. Given the importance of maintaining good oral health, an increase in services, which improve the community based geriatric care culture, are needed. Future health professionals, during their early training years, should pay visits to nursing homes to mix with the residents and better their relationship with the elderly residents. Improved funding and resources should be considered to prioritize the elderly for oral health promotion and screening programmes, identifying them as a group that are currently of seemingly low priority, whose longevity and improved quality of life, morbidity and mortality may be significantly enhanced with provision of education and support to both the elderly and their care teams. Community based oral health care should become compulsory for nursing homes. Input from nutritionists should be considered to support healthier diets for the elderly that improve oral health.

## Conclusion

Maintaining healthy oral and GI tract microbiomes is of great benefit to the host. It appears that the oral dysbiotic biofilm contributes to factors that initiate pathogenesis of disease both locally and at distant organ sites remote from the GI tract. The mechanism by which oral pathogens cause destruction is via subversion of immune responses and by differential inflammatory signaling pathway activation. The GI tract dysbiotic microbes appear to display features related to behavior. The cross-links involve the endocrine pathway proteins, such as GABA, serotonin, stress hormones, along with various others. This is because GI tract microbiome bacteria are enriched in neurochemicals and are involved in local upkeep through sensory vagus nerve signaling pathways with the brain. One explanation may be that the loss of neurochemicals through a changing microbiome consortium cannot feedback, the information to the brain to keep control over ongoing inflammation. These balances need to be maintained by overcoming nutritional deficits that life styles, especially those associated with the progress of aging, may have introduced into daily life.

The current article discusses nutritional factors, including the benefits of adopting a calorically appropriate, low-carbohydrate, adequate protein and fat containing diet which appear to be the best way to prevent and/or delay the progression of both periodontal disease and AD. Virgin coconut oil and MCTs supplements provide ketone bodies which can support the brain, and help to improve oral microbiota. The current article also discusses multiple shared risk factors of periodontitis and AD in relation to their co-morbid status development. One important dental aspect highlighted at least a decade ago, but taken little notice of, is how fewer teeth alter people’s eating habits and their dietary choices, which impacts on oral, metabolic, GI tract and brain health. In the context of a multifactorial intervention (MEND™ programme), a lower carbohydrate diet supplemented with virgin coconut oil or MCT-Oils, fish or omega-3 fatty acids, selected nutrients or phytochemicals along with, for example, improvements in oral hygiene and sleep were necessary to improve oral health. These have already shown promise in delaying and even reversing symptoms of mild cognitive decline and AD (Bredesen, 2014; Bredesen et al., 2016). In conclusion, more research is needed to evaluate the effectiveness of diet-based interventions in supporting oral health, and to assess the potential impact of nutrition on AD onset and progression.

## Author Contributions

AH initiated the review text. UG contributed to dietary aspects. SJR contributed to the sleep sections, and SKS contributed to the overall focus and provided editorial help for the compilation of the article as PhD supervisor of AH. SC provided critical feedback and funding.

## Conflict of Interest Statement

The authors declare that the research was conducted in the absence of any commercial or financial relationships that could be construed as a potential conflict of interest.
